# Generation of the First TCR Transgenic Mouse with CD4^+^ T Cells Recognizing an Anti-inflammatory Regulatory T Cell-Inducing Hsp70 Peptide

**DOI:** 10.3389/fimmu.2016.00090

**Published:** 2016-03-09

**Authors:** Manon A. A. Jansen, Martijn J. C. van Herwijnen, Peter J. S. van Kooten, Aad Hoek, Ruurd van der Zee, Willem van Eden, Femke Broere

**Affiliations:** ^1^Department of Infectious Diseases and Immunology, Utrecht University, Utrecht, Netherlands

**Keywords:** heat shock protein 70, transgenic mouse, autoimmunity, hybridoma, regulatory T cells

## Abstract

Antigen-specific regulatory T cells (Tregs) directed at self-antigens are difficult to study since suitable specific tools to isolate and characterize these cells are lacking. A T cell receptor (TCR)-transgenic mouse would generate possibilities to study such ­antigen-specific T cells. As was shown previously, immunization with the mycobacterial heat shock protein (Hsp) 70-derived peptide B29 and its mouse homologs mB29a and mB29b induced anti-inflammatory responses. Furthermore, B29 induced antigen-­specific Tregs *in vivo*. To study mB29b-specific Tregs, we isolated the TCR from T cell hybridomas generated against mB29b and produced a TCR transgenic mouse that expresses a MHC-class II restricted mB29b-specific TCR. These TCR transgenic CD4^+^ T cells were found to cross-react with the B29 epitope as identified with peptide-induced proliferation and IL-2 production. Thus, we have successfully generated a novel mouse model with antigen-specific CD4^+^ T cells that recognize self and bacterial Hsp 70-derived peptides. With this novel mouse model, it will be possible to study primary antigen-specific T cells with specificity for a regulatory Hsp70 T cell epitope. This will enable the isolation and characterization CD4^+^CD25^+^ Tregs with a proven specificity. This will provide useful knowledge of the induction, activation, and mode of action of Hsp70-specific Tregs, for instance, during experimental arthritis.

## Introduction

Heat shock protein (Hsp) 70 is a ubiquitously expressed protein and plays a role as chaperone in protein folding, either after protein synthesis or under conditions of cellular stress (1, 2). Hsp70 is evolutionary conserved and is expressed by many species, including bacteria and vertebrates. This is reflected by a high degree of homology of Hsp between species. Interestingly, Hsps are also highly immunogenic, which might be explained by the fact that Hsps are found in bacteria that surround us. However, not only Hsp-derived peptides from bacteria are immunogenic but also peptides derived from self-Hsp can trigger immune responses (3, 4). Peptides derived from endogenous Hsp70 can not only be found in MHC class I molecules but are also known to be present in MHC class II (5–8). This indicates that Hsp70-derived peptides can be recognized by immune cells in a MHC class II-dependent manner. This is supported by experiments in which CD4^+^ T cell responses against Hsp70-derived epitopes have been identified after immunization with bacterial Hsp70 (7). Apart from presentation during cellular homeostasis, endogenous Hsp can also be presented in MHC when it is upregulated during cellular stress such as heat shock (8, 9). Due to the high degree of homology of Hsp between species, cross-reactive responses occur in which foreign Hsp-peptide reactive T cells can recognize self-Hsp peptides (7, 8, 10, 11).

Interestingly, the administration of Hsp70 can result in anti-inflammatory responses, which has been shown by the suppression of disease in animal models for chronic inflammation, due to activation of Hsp70-specific regulatory T cells (Tregs) that are cross-reactive with self-epitopes of Hsp70 (4, 7). Tregs are a subset of specialized CD4^+^ T cells with high suppressive potential and are therefore important targets for immune modulation of inflammatory diseases (12). Therefore, activating these cells *via* Hsp peptides is a growing field of interest, especially in inflammatory diseases in which the disease-inducing antigens are unknown.

Previously, we have shown that mycobacterial Hsp70 peptide B29 is highly conserved and immunogenic. Immunization with, or intranasal administration of B29, activates B29-specific Treg *in vivo*, which are potent suppressors of experimental arthritis (7). Several tools, such as T cell lines and T cell hybridomas, have been used to study Hsp-specific T cell responses in the past. For instance, T cell lines generated from mycobacterial Hsp60 immunized rats specific for a highly conserved sequence of Hsp60 have been used to study the suppressive potential of Hsp-specific T cells (10). Similar results were obtained with T cell lines generated from mycobacterial Hsp70 immunized rats: Hsp70-specific T cells reduced the severity of arthritis in the experimental arthritis model (13). However, none of these systems allows the evaluation of primary T cell responses (*in vivo)*, the behavior of Hsp70-specific Tregs upon activation, or the induction of particular T cell subsets such as effector T cells and Tregs after administration of Hsp70. Especially, antigen-specific Tregs are difficult to study since these cells comprise only a small fraction of the total T cell population and are difficult to culture and maintain *in vitro* (14).

Therefore, we aimed to generate a mouse model to study primary and naive Hsp70-specific CD4^+^ T cells in more detail. For that reason, we isolated the T cell receptor (TCR)-α and TCR-β chain genes from a T cell hybridoma generated against peptide mB29b, a mammalian homolog of B29 (Table 1). This hybridoma was found to cross-react with B29 and another mammalian homolog: mB29a (9). With the TCR-α and TCR-β chain genes, we generated a TCR transgenic mouse with Hsp70 peptide-specific CD4^+^ T cells. We show that CD4^+^ T cells from the mB29b-TCR transgenic mouse undergo antigen-specific proliferation and produce IL-2 after restimulation with B29 or its mouse homologs. In future studies, primary CD4^+^ T cell responses directed against self and bacterial Hsp70 peptides can be investigated *in vitro* and *in vivo*. Particularly, the activation and differentiation of antigen-specific CD4^+^ Tregs can be studied with this model, which are not possible with long-term T cell lines or T cell hybridomas.

**Table 1:** **Amino acid sequences of Hsp70 peptides**.

Peptide	Sequence	Protein	Origin
mB29a	VLRVINEPTAAALAY	Hspa9 (GRP75)	*Mus musculus*
			*Homo sapiens*
mB29b	VLRIINEPTAAAIAY	Hspa1a, Hspa8	*Mus musculus*
			*Homo sapiens*
a1a-long29	DAGVIAGLNVLRIINEPTAAAIAYGLDRTGK	Hspa1a (Hsp72)	*Mus musculus*
			*Homo sapiens*
a8-long29	DAGTIAGLNVLRIINEPTAAAIAYGLDKK	Hspa8 (Hsp70)	*Mus musculus*
			*Homo sapiens*
B29	VLRIVNEPTAAALAY	DnaK (Hsp70)	*Mycobacterium tuberculosis*

*The amino acid sequence and origin of the Hsp70-derived peptides used in this study*.

## Materials and Methods

### Mice, Peptides, and Antibodies

Female Balb/c mice aged 8–12 weeks were purchased from Charles River and used as cell donors to create hybridomas and as source of APCs for coculture assays. Animals were kept under standard conditions at the animal facility, and all experiments were approved by the Animal Experiment Committee of Utrecht University. Mice strains used for the generation of the mB29b-TCR transgenic mouse were F1 of (CBA × C57BL/6) mice (Charles River). Hsp70-derived peptides (mB29a, mB29b, B29) were identified previously (7) and were obtained from GenScript Corporation. The amino acid sequences and origin of the peptides are shown in Table 1. Anti-MHC-II (I-Ad/I-Ed) antibody (clone M5/114) 5 μg/ml; gift from Louis Boon from Bioceros B.V., Utrecht, The Netherlands was used to block ­MHC-II-peptide TCR interactions in cocultures. To stain mB29b-specific cells, an APC-labeled murine – mB29b-specific tetramer that is composed of mB29b [VLRIINEPTAAAIAY linked to I-A(d)(BALB/c haplotype-matched MHC class II molecule)] was used for 90 min at 20 μg/ml. This tetramer is a gift from NIH Tetramer Core Facility (Emory University, Atlanta, GA, USA).

### Generation of mB29b-TCR Hybridoma (LHEPs)

CD4^+^ T cell hybridomas (named LHEPs) were generated against Hsp70 peptide mB29b in our laboratory, as described previously (9). Activation of specific hybridoma clones by mB29b was addressed by incubating hybridoma cells (2 × 10^4^/well) with irradiated (10,000 rad) A20 B lymphoma cells as APC (2 × 10^4^/well) loaded with Hsp70 peptides in 96 wells flat bottom plates for 48 h and pulsed with ^3^H-thymidine (0.4 μC/well) for an additional 16 h to measure activation-induced cell death (AICD).

As a positive control, hybridomas were stimulated with 2 μg/ ml ConA. After 48 h coculture, supernatants were harvested and frozen at −20°C. IL-2 production by hybridomas was studied by culturing the harvested supernatants with IL-2 responder ­CTLL-16 cells (cytotoxic T cell line; 5 × 10^3^/well) for 24 h. The CTLL-16 cells were pulsed with ^3^H-thymidine (0.4 μC/ well) for another 16 h (Amersham Biosciences Europe GmbH, Roosendaal, The Netherlands). The 5/4E8 hybridoma specific for proteoglycan (PG)-derived peptide PG70-84 (15) was used as a control. RNA from the mB29b-TCR hybridoma was sequenced to determine the TCR usage. Sequencing revealed that the isolated mB29b TCR consisted of Vα 7 (TRAV-7.01-J26.01 in the IMGT nomenclature) and Vβ 8.2. The Vβ 8.2 is according to the NCBI nomenclature, this correlates with TRBV-13-2.01-D2.01-J2-7.01 in the IMGT nomenclature.

### MHC Restriction of T Cell Hybridomas

The MHC restriction of the peptide mB29b-specific hybridomas was determined using mouse anti-I-Ad (BD PharMingen) mAb. Hybridoma cells were cultured with A20 APCs in the presence of 0.5 μg/ml peptide mB29b and anti-I-Ad mAb (MKD6) at a concentration of 20 μg/ml. The effect of mAb on IL-2 production was determined with the CTLL-16 bioassay, as described above.

### Cloning of the αβ TCR

Total RNA was isolated from the mB29b-TCR hybridoma cells by extraction with RNAzol (Invitrogen). The oligo(dT)_12–18_ primer from the Superscript Reverse Transcription kit (Invitrogen) was used for reverse transcription of the isolated RNA. To express the TCR mB29b genes in the transgenic mice, we cloned the Vα and Vβ genes into pTα and pTβ cassettes obtained from Kouskoff et al. (16). Isolation of genomic DNA from the mB29b-TCR hybridoma was performed to obtain full length rearranged TCRα and TCRβ DNA, including leader and intron sequences. mB29b-TCR DNA was amplified by PCR using the primer for the Vα chain (Forward: TRAV7-1-Xmal: *TAAT*CCCGGG*AGAATGAAGTCCTTGTGTGTTTCAC*, Reverse; TRAJ26.01-Notl: *TAAT*GCGGCCGC*ACAGTAGACCTCAGGTCCCCCTCAC*) and the Vβ chain (Forward: TRBV13-2.-1-Xhol:*TAAT*CTCGAG*AAGATGGGCTCCAGGCTCTTC*; Reverse:TRBJ2-7.01-*Sac*II: *TAAT*CCGCGG*CCTGGTCTACTCCAAACTACTCC*). The PCR products of the two fragments were cloned using TA overhang into the pGEM-T easy vector (Promega). The constructs were subsequently introduced into *E. coli* DH5α. The *Xma*I and *Not*I released DNA fragment, containing the TCRα chain, was cloned into the pTα cassette. The *Xho*I and *Sac*II DNA fragment, containing the TCRβ chain, was cloned into the pTβ cassette. Both were transfected into XL10 gold cells (Stratagene) by electroporation.

### *In Vitro* Expression of the αβ TCR

The pTα cassette, the pTβ cassette, and the pcDNA3 plasmid (containing neomycin resistance gene) were electroporated into the mouse 58α^−^β^−^ T cell hybridoma that lacks functional TCR chains (17). Transfected cells were cloned using limiting dilution in 96 wells plates using the FACS Vantage (BD) and cell lines were cultured in the presence of Geneticin 418 (0.8 mg/ml). PCR was used to validate DNA incorporation and transfected cells were tested for antigen specificity in a similar manner as the ­hybridomas (described above).

### Generation of the mB29b-TCR Transgenic Mouse

T cell receptor transgenic mice were generated in our laboratory, as described previously (15, 17, 18). The pTα mB29b-TCR and the pTβ mB29b-TCR plasmids were linearized using *Sal*I (pTα) and *Knp*I (pTβ). *Via* pronuclear injection a mixture of the plasmids were introduced into fertilized eggs of F1 (CBA × C57BL/6) mice. Two mB29b-TCR transgenic founders were identified by PCR analysis of genomic DNA (same primers as described above). Founder 2 was mated with Balb/c mice (Balb/cBYJRj; Jackson laboratories), and offspring was tested for peptide specificity, as described below.

### Measurement of Antigen-Specific T Cell Responses from mb29b-TCR Mice

Blood was taken from founders and depleted from erythrocytes with ACK lysis buffer (H_2_O containing 150 mM NH_4_Cl, 10 mM KHCO_3_, and 0.1 mM EDTA, pH 7.2–7.4). Blood cells (founder 1: 1 × 10^5^, founder 2: 5 × 10^5^, depending on cell yield after blood collection) were cultured for 96 h with 1 × 10^6^ irradiated A20 cells as APCs. Cells were stimulated with 2 or 20 μg/ml B29 or with 5 μg/ml ConA as a positive control. Peripheral blood lymphocytes (PBLs) from founders were tested for antigen-specific responses to 2 or 20 μg/ml mB29a, mB29b, or B29 peptides. Proliferation was determined by ^3^H-thymidine incorporation during the final 16 h of culture, and IL-2 production was determined by Luminex. Splenocytes from offspring were screened for the expression of TCRα and TCRβ chain. The mB29b-TCR positive splenocytes were also tested for antigen specificity.

### Flow Cytometric Analysis

Single cell suspension of splenocytes, lymph node cells, or thymocytes were made, and these were stained with antibodies CD3-APC (OKT-3, BD Biosciences), CD4-V450 (RM4-5, eBioscience), CD8-V500 (RPA-T8, BD Biosciences company), Vβ8-PE (F23.1, BD Biosciences) KI-67-PerCp-Cy5.5 (BD56, BD biosciences), CD25-PerCp-Cy5.5 (PC61.5, Ebioscience), IFN-γ-FITC (XMG1.2, BD biosciences), CD44-APC (IM7, ebioscience), CD62L-FITC (MEL-14, BD biosciences) or FoxP3-eFluor450 (FJK-16s, ebioscience) and incubated for 30 min at 4°C. Cells were washed three times with PBS containing 2% FCS. Cells were acquired on the FACS Canto II (BD) and analyzed with FlowJo 7 (Tree Star). For cell activation experiments, splenocytes from transgenic mice or littermates were cultured (1 × 10^5^ cells/well) for 24 h in the presence of 20 μg/ml mB29b, in which the last 4 h was in the presence of 1 μg/ml Brefeldin A.

### Histology

For histology, thymus, spleen, inguinal lymph nodes (iLN; representative draining LNs), and liver were isolated from mB29b-TCR positive mice, or negative littermates. Tissues were fixed in 10% neutral buffered formalin, embedded in paraffin, and 5 μm saggital sections were stained with hematoxylin and eosin (H&E). Immunohistochemistry was performed to T cells and general proliferation in lymphoid tissues. Briefly, cryosections (5 μm) were fixed in ice-cold acetone and blocked against endogenous peroxidase with 0.3% hydrogen peroxide in methanol. Non-specific staining was blocked with a 1% BSA solution, and sections were incubated with primary antibodies against CD3 (BD Biosciences) or Ki-67 (BD Biosciences). Secondary staining was performed with an anti-rat HRP antibody (Millipore), and Peroxidase activity was developed using the DAB Peroxidase Substrate kit (Vector Laboratories). Sections were counterstained with hematoxylin and mounted with Aquatex (Merck, Darmstadt, Germany). Pictures were taken using an Olympus BX41 microscope and analyzed with Cellsens entry 1.9 software (Olympus Corporation).

### Statistics

Unless stated otherwise, data are expressed as mean ± SD. Statistical analyses were carried out using Student’s *t*-test or the two-way ANOVA test using Prism software (Version 6.05). *p* ≤ 0.05 was considered significantly different.

## Results

### MHC Class II Restricted Recognition of Hsp70 Peptides mB29a, mB29b, and B29 by mB29-TCR Hybridoma LHEP4

**Figure 1:**
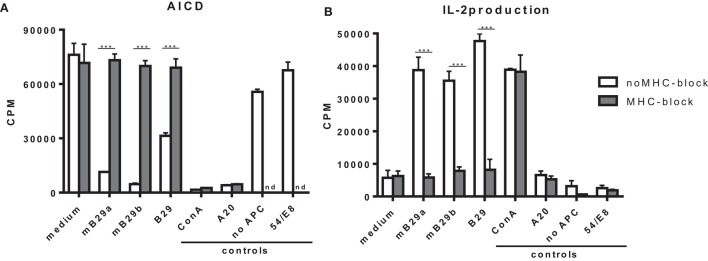
**The hybridoma LHEP4 recognizes Hsp70 peptides mB29a, mB29b, and B29 in an MHC class II restricted way**. Splenocytes from BALB/c mice immunized with mB29b were fused with BW5147 cells, which resulted in mB29b-specific CD4^+^ T cell hybridoma clones (LHEPs). **(A)** Activation-induced cell death (AICD) is measured by reduced thymidine incorporation upon activation with mB29a, mB29b, and B29. MHC-II blockage results abrogate AICD, which indicate that the mB29b-TCR hybridomas are MHC-II restricted. **(B)** IL-2-dependent CTLL-16 proliferation was measured to demonstrate that supernatant of activated hybridomas as depicted in **(A)** produce IL-2. As shown in **(B)**, IL-2 production is present after stimulation with mB29a, mB29b, and B29. MHC-II blockage results in a diminished IL-2 production. As a positive control, cells were stimulated with ConA. As a negative control, LHEP4 hybridoma cells were cultured in medium only, in the presence of mB29b (no APC). Additional controls included A20 cells cultured with mB29b, or the 5/4E8 hybridoma cultured in the presence of mB29b peptide. ^3^H-thymidine incorporation is shown as mean of triplicate samples/well ± SEM. Data shown are representative of three independent experiments.

T cell hybridomas specific for Hsp70 peptide mB29b were generated by immunizing BALB/c mice with the peptide mB29b and fusing splenocytes from these mice with BW5147 cells followed by limiting dilution to obtain individual clones of the mB29b-TCR hybridomas. In order to test the TCR specificity of the generated hybridomas, which we named LHEPs, the cells were cultured in the presence of A20 cells as APCs and peptide mB29b, or its homologs mB29a and B29 (Figure 1). AICD and IL-2 production (9), two characteristics of an activated hybridoma, were measured for seven LHEPs. The AICD was determined by ^3^H thymidine incorporation, and the IL-2 production was measured by IL-2-dependent proliferation of the CTLL-16 cell line. Data of LHEP4 are depicted as a representative example in Figure 1. As seen in Figure 1, stimulation of LHEP4 with mB29a, mB29b, and to some extend with B29 resulted in cell death and IL-2 production. To determine whether recognition of Hsp70 peptides was MHC class II restricted, LHEPs were stimulated with peptide in the presence of an MHC class II blocking antibody. In this case, no AICD and a strongly reduced IL-2 production was seen upon stimulation with Hsp70 peptides, indicating that peptide recognition of mB29b-TCR hybridomas was indeed MHC class II restricted (Figures 1A,B). The mB29b peptide was presented in the context of I-Ad molecules, since a mAb against I-Ad (MKD6) completely abrogated the mB29b-specific *in vitro* proliferation of the LHEP4 hybridoma (data not shown).

Screening of the obtained selected mB29b-specific T cell hybridomas revealed seven LHEPs in total that were responsive to mB29b, of which five were cross-reactive to mB29a, and four that were cross-reactive to B29.

### LHEPs Respond to Several Length Variants of the Hsp70 Peptide

**Figure 2:**
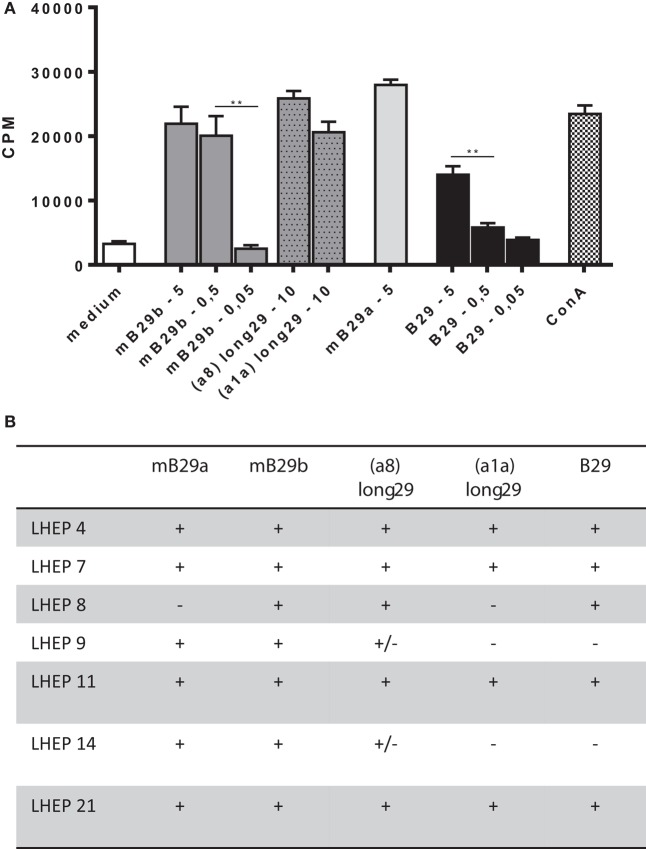
**The mB29b-TCR hybridoma LHEP4 produces antigen-specific IL-2 upon coculture with primary antigen-presenting cells**. **(A)** LHEP4 was cocultured with irradiated splenocytes loaded with different concentrations [ranging from 5, 0.5, to 0.05 μg/ml, as well as 10 μg/ml length variants of mB29b (hspa8 = Hsc70 or hspa1a = Hsp72)] (20) of Hsp70 peptides as indicated, after which supernatants were collected. CTLL-16 cell cultures were supplemented with supernatants from these stimulations to determine IL-2-dependent proliferation of CTLL-16 cells. As a control, cocultured LHEP4 cells were unstimulated, or stimulated with ConA. ^3^H-thymidine incorporation is shown as mean of triplicate samples/well ± SEM. Data shown are representative of three independent experiments. **(B)** mB29-TCR hybridomas were stimulated with Hsp70 peptides mB29a, mB29b, or B29, as well as length variants of mB29b [hspa8 = Hsc70 or hspa1a = Hsp72 (20), as described in **(A)**]. The + symbol (cpm >15,000) indicates AICD and IL-2 production upon coculture with supernatants from peptide-stimulated LHEPs. Weak responses are depicted as ± (on average the cpm = 4000). Medium stimulated samples show on average a cpm of 1500. Data shown summarize three independent experiments.

We next determined a dose response of LHEP4 to the Hsp70 peptides, as well as recognition of specific length extension variants of mB29b to assess the response to processed peptides. For this, LHEP4 was cocultured with irradiated splenocytes in the presence of the extended hspa8 (=Hsc70) or hspa1a (=Hsp72) (19) variants of the mB29b peptide, later referred to as a8-long29 and a1a-long29. CTLL-16 proliferation induced by IL-2 production from LHEP4 indicated that these length variants could be recognized (Figure 2A). Although IL-2 production decreased in cultures in which LHEP4 was stimulated with low amounts of mB29b or B29 (data not shown), CTLL-16 proliferation remained detectable, indicating that LHEP4 responds sensitively to presented Hsp70 peptides (Figure 2A).

Apart from LHEP4, the previously selected T cell hybridomas were screened for the recognition of Hsp70 peptide (length variants) (Figure 2B). All LHEPs responded to the mB29b peptide when presented by different primary APC, indicating that the mB29b-TCR hybridomas recognize Hsp70 peptides presented by APCs from various sources (data not shown). As was previously seen (Figure 2B), not all LHEPs were cross-reactive to other peptides, including the length variants of mB29b (hspa8 or hspa1a). All mB29b-TCR hybridomas that recognize different length variants of mB29b provide a broad recognition spectrum.

### Cloning of the TCRα and TCRβ Chain from mB29b-TCR Hybridoma LHEP4 into TCR^−^ Cells Results in Hsp70 Peptide-Specific Transfectants

**Figure 3:**
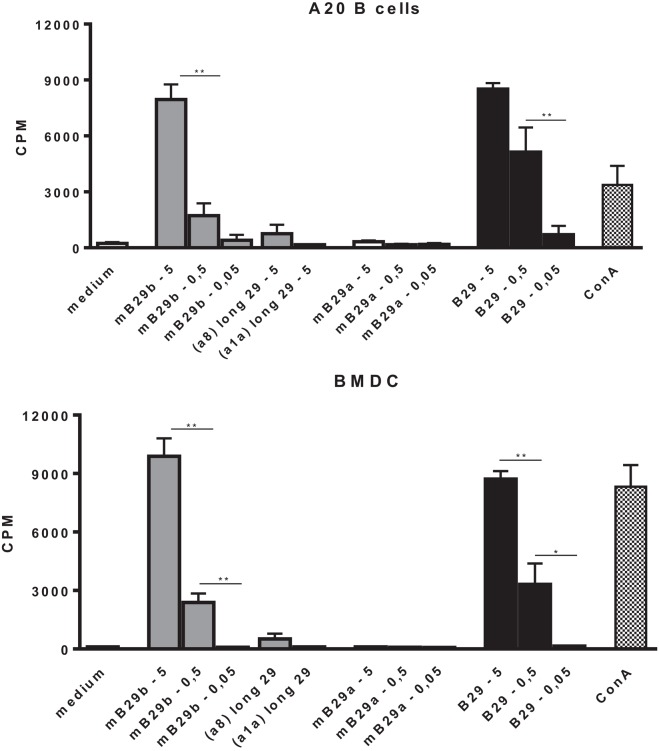
**Cloning of the TCRα and TCRβ chain from mB29b-TCR hybridoma LHEP4 into TCR^−^ cells results in Hsp70 peptide-specific transfectants**. Cloned transfected cells were cocultured with A20 B cells **(A)** or BMDCs **(B)**, which were loaded with 5, 0.5, or 0.05 μg/ml Hsp70 peptides. After stimulation with Hsp70 peptides mB29a, mB29b, or B29, as well as length variants of mB29b (a1a or a8), supernatants were added to CTLL-16 cells and IL-2-dependent proliferation was determined after 24 h. The graph shows more IL-2-dependent proliferation of CTLL-16 cells when stimulated with a high concentration Hsp70 peptide mB29b or B29. There is no response to stimulation with mB29a and to a minor extent to the length variants of mB29b (a8 or a1a). Similar results were obtained with the BMDC coculture **(B)**. As a control, cocultured LHEP4 cells were unstimulated or stimulated with ConA (data not shown). ^3^H-thymidine incorporation is shown as mean of triplicate samples/well ± SEM. Data shown are representative of two independent experiments.

Based on the specificity and the strong cross-reactive responses of LHEP4, the TCRα and TCRβ chain of this mB29b-TCR hybridoma were cloned into TCR expression vectors, which were transfected into cells lacking the TCR (TCR^−^). The transfected cell line was cocultured with BMDC or irradiated A20 B cells, which were loaded with Hsp70 peptides to confirm the antigen specificity of the transfectant. Results showed a dose-dependent response to mB29b and B29, but not to mB29a (Figure 3). Irradiated thymocytes and splenocytes were also used as APC and gave similar proliferative responses (data not shown). Furthermore, addition of shorter variants of the mB29b peptide to LHEP4 and the transfected cells, failed to stimulate the mB29b-specific hybridoma and cells indicating the specificity of the hybridoma and transfected cells (data not shown). Together, these data confirmed that the TCRα and TCRβ chain were successfully cloned into TCR expression vectors, and therefore we transferred the TCRα and TCRβ chain constructs to mouse oocytes *via* pronuclear injection.

### Antigen Recognition of Cells from mB29b-TCR Transgenic Mouse

**Figure 4:**
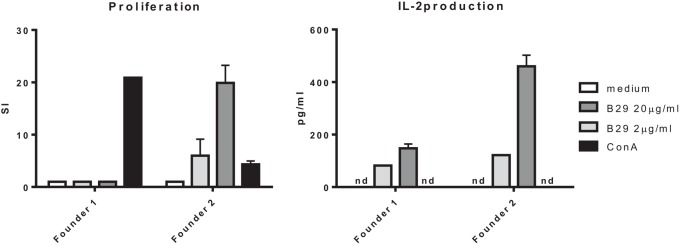
**Antigen recognition by mB29b-TCR transgenic mouse**. PBLs from two founders, positive for the TCRα and TCRβ chain, were cultured for 96 h with irradiated A20 B cells and stimulated with 2 or 20 μg/ml B29, or 5 μg/ml ConA as a positive control. PBLs from founder 2 proliferated in a dose-dependent response. The PBLs from founder 1 did not show any proliferation after B29 stimulation. The PBLs from both founders produced IL-2 but the PBLs from founder 2 produced higher amounts. Proliferation was determined by ^3^H-thymidine incorporation during the final 16 h of culture and IL-2 production was determined by Luminex. Data are from one experiment.

After pronuclear injection of the DNA expressing TCRα and TCRβ into the donor zygote, the zygote was injected in a foster mouse from which several pups were born that had incorporated the constructs. From these mice, two founders were positive for both constructs with PCR (data not shown). PBLs from the two founders were stimulated with B29 in a coculture with irradiated splenocytes. PBLs from one founder proliferated and produced IL-2 in response to B29 stimulation (Figure 4). Next, the positive founder was mated with Balb/c mice and F1 mice were screened for the expression of the TCRα and TCRβ chain and splenocytes from mB29b-TCR positive offspring were tested for antigen specificity and compared to negative littermates. We observed responses to mB29b, B29, and mB29b length variant (a8) long29 (Figure 2B), while mB29b-TCR negative littermates showed no response to any of the peptides tested (data not shown). These data show that we successfully generated mB29b-TCR transgenic mice with cells with a functional TCR that recognized Hsp70 peptides.

### Flow Cytrometric Analysis of mB29-TCR Transgenic Mouse Tissues

**Figure 5:**
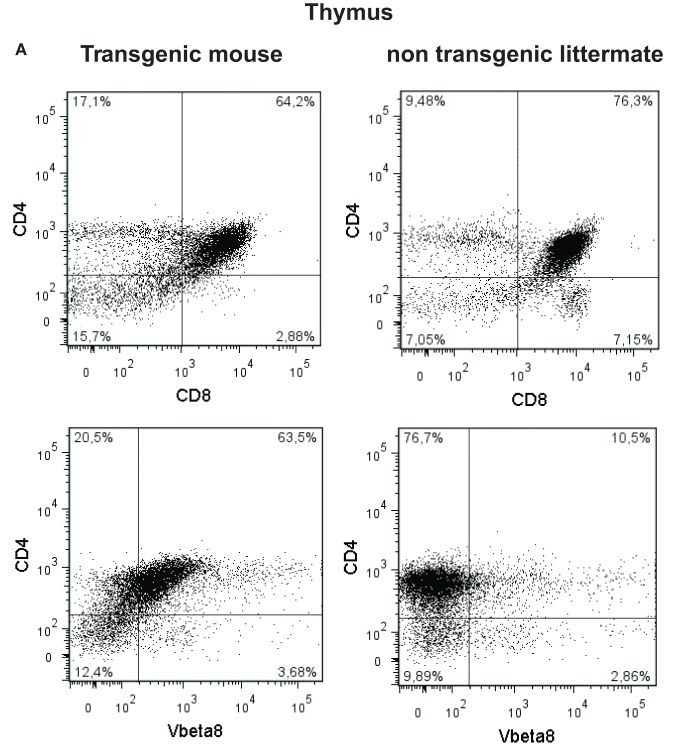

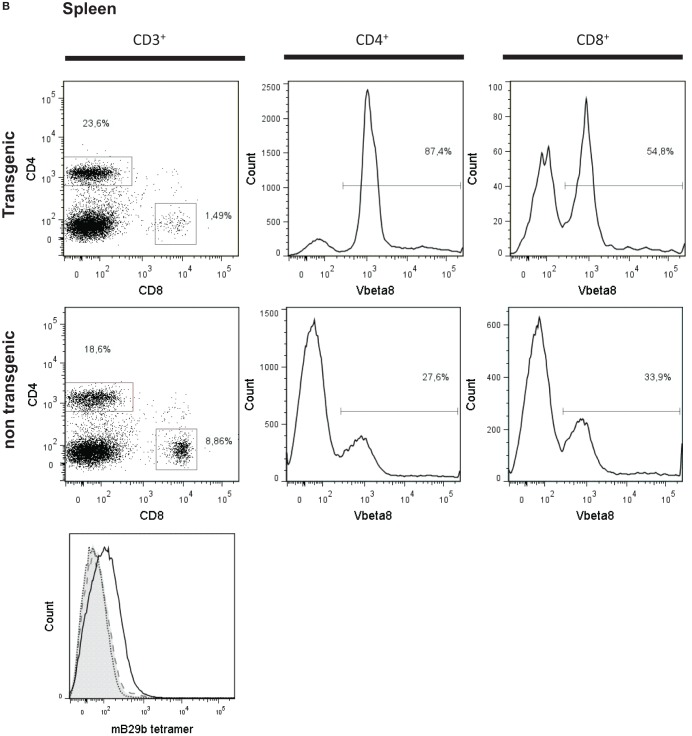

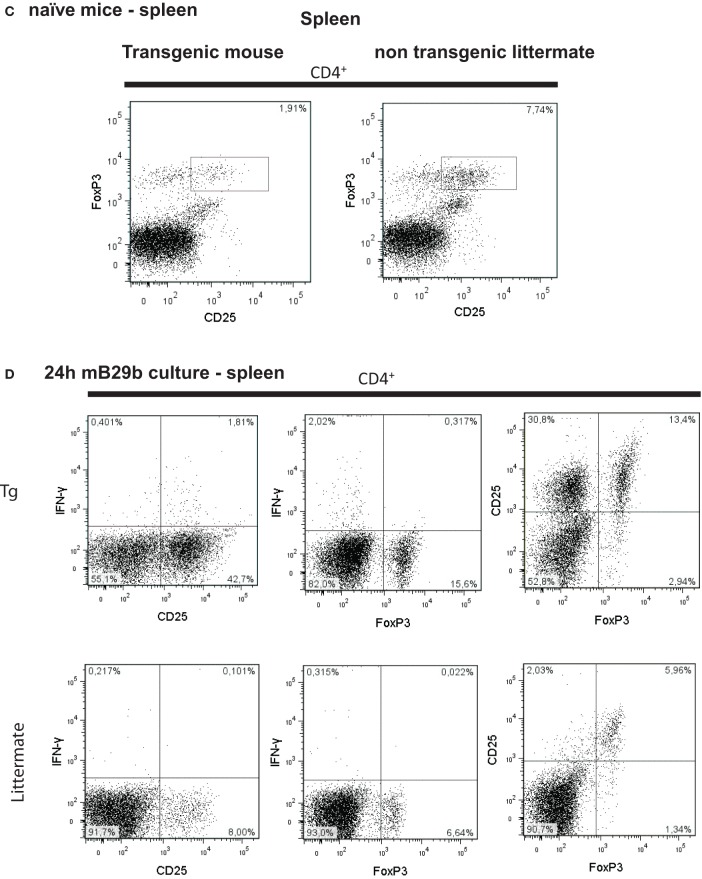
**CD4^+^ and CD8^+^ T cell distribution in tissues of mB29-TCR transgenic mouse**. **(A)** Single cell suspensions were made from thymus of mB29b-TCR transgenic mice or negative littermates. Cells were stained for the expression of CD3, CD4, CD8, and Vβ8. Live cells were gated on the forward scatter (FSC) and side scatter (SSC) and the percentage of CD4^+^ and CD8^+^ cells of the live cells are depicted in the upper panel of **(A)**. In the lower panel of **(A)**, the percentage of CD4^+^Vβ8^+^ of all live cells is shown. The different CD4^+^ and CD8^+^ T cell distribution of the mB29b-TCR transgenic mouse compared to non-transgenic littermates is due to the transgenic background, in which formation of T cells is changed. Plots shown are representatives of three independent experiments. **(B)** Distribution of CD4^+^ and CD8^+^ cells in the CD3^+^ cells (left row of graphs), histograms of CD4^+^ cells (middle row of graphs) and CD8^+^ cells (right row of graphs) of total CD3^+^ cells are depicted. The transgenic mouse shows an increase in CD8^+^Vβ8^+^ and CD4^+^ Vβ8^+^ T cells compared to the non-transgenic littermate. The overlay histogram at the bottom of **(B)** represents the amount of mB29b-specific CD4^+^ T cells in a WT Balb/c (filled gray), a negative littermate (dashed line), and an mB29b TCR Tg mouse (black line). The dotted line shows splenocytes stained with a CLIP tetramer as negative control. Each line represents three different mice. **(C)** CD25 and FoxP3 expression measured within de CD4^+^ population of splenocytes derived from naive mice. **(D)** CD25, IFN-γ, and FoxP3 markers are measured by flow cytometry upon 24 h culture of splenocytes from both negative littermates as transgenic mouse in the presence of mB29b.

Next, we examined the presence of CD3^+^, CD4^+^, CD8^+^ T cells in thymus of the mB29b-TCR transgenic mouse. Since the mB29b-TCR hybridoma was recognized by the antibody directed against Vβ8, we also screened the thymus for Vβ8^+^ T cells. The transgenic mice and littermates had a similar frequency of CD4^+^ cells, whereas the transgenic mouse had an increased percentage of Vβ8^+^ T cells in the thymus and a decreased amount of CD8^+^ cells (Figure 5A). In the spleen, the same differences in CD4^+^ T cell and CD8^+^ T cell distribution (more CD4^+^ T cells compared to a wild type mouse) were observed, as well as an increased ­number of Vβ8^+^ T cells were detected (Figure 5B). This resulted in changes in the CD4:CD8 ratio in both the thymus and spleen. The increased CD4:CD8 ratio became more evident in later generations. We observed a similar distribution and percentage of T cells in the LN (data not shown). To test the specificity of the CD4^+^ T cells in the mB29b-TCR Tg mouse, we stained splenocytes with an APC-labeled murine – mB29b-specific tetramer that is composed of mB29b [VLRIINEPTAAAIAY linked to I-A(d)] and also cultured splenocytes in the presence of mB29b for 24 h. As shown in the lower part of Figure 5B, the mB29b TCR Tg mouse contains more tetramer-specific CD4^+^ T cells than the WT Balb/c and the negative littermate. Figure 5D demonstrates that after 24 h of culture in the presence of mB29b, more CD4^+^ T cells are activated (IFN-γ and CD25 expression) in the spleen of the transgenic mouse, in comparison with the negative littermate. Furthermore, also the CD4^+^CD25^+^FoxP3^+^ population is more pronounced in the mB29b TCR Tg mouse compared to the negative littermate, while in naive mice, FoxP3 expression is lower in mB29b Tg mice (Figure 5C).

### mB29b-TCR Transgenic Mice Show an Increase in Naive Cells

**Figure 6:**
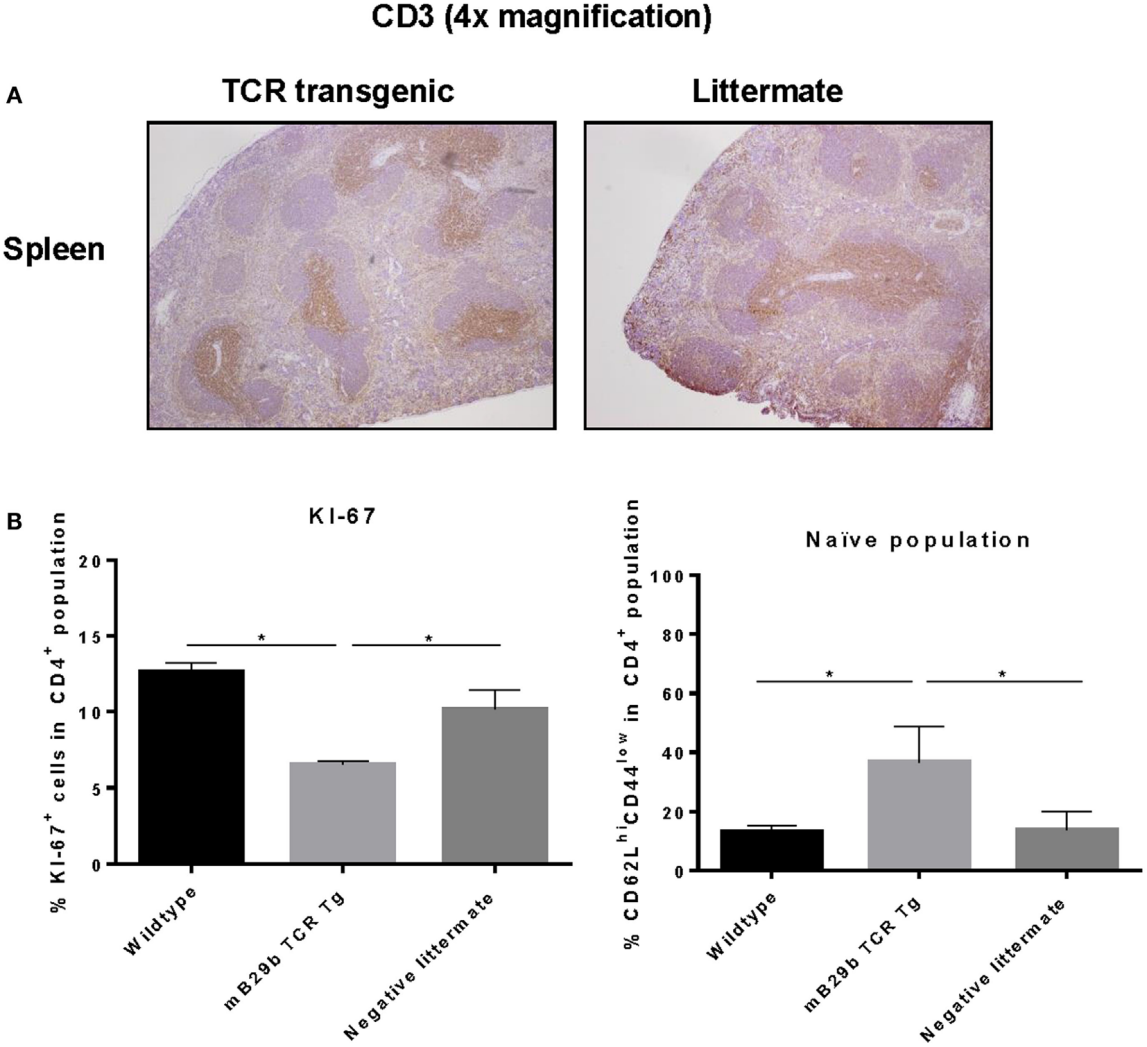
**mB29b-TCR transgenic mouse shows no pathological changes in histology**. **(A)** Thymus, inguinal lymph nodes (iLN), and liver (data not shown) were isolated from mB29b-TCR positive mice, or negative littermates. **(A)** shows sections of the spleen in which no structural changes were observed between the mB29b-TCR transgenic mouse and the non-transgenic littermate. Immunohistochemistry was performed to T cells (αCD3) and general proliferation in lymphoid tissues. The distribution of CD3^+^ T cells in the mB29b-TCR transgenic mouse does not deviate from the non-transgenic littermate. Pictures show the spleen in a 4× magnification. **(B)** Splenocytes were isolated from naive wild type Balb/c, mB29b TCR Tg mice and negative littermates and CD4^+^ cells were stained for KI-67 or CD44 and CD62L. Data are obtained by flow cytometry. Data are shown as mean of triplicate samples/well ± SEM. Data shown are representative of two independent experiments.

To investigate cell distribution and activation, both histology and flow cytometry were used. Tissue sections from thymus, spleen, iLN, and liver were made and stained for H&E or CD3 (Figure 6). Based on the H&E stained tissue slides, no apparent changes are observed in iLN, spleen, thymic, or liver tissue architecture (data not shown). In addition, the distribution of CD3^+^ T cells in the different lymphoid tissues was comparable between the TCR transgenic mouse and the littermate (Figure 6A). However, we did find a difference in proliferative activity, as based on the Ki-67 expression in splenocytes (Figure 6B). The KI-67 positive population is reduced in the mB29b TCR Tg mouse when compared to the wild type mice and negative littermates. This correlates with the enhanced non-proliferative naive cell population (defined as CD62L^hi^CD44^low^) in naive mB29b TCR Tg mice (Figure 6B). Overall, histological analysis revealed no major differences in T cell distribution or activation between the founder and control littermates in lymphoid organs, suggesting no development of gross pathology.

## Discussion and Conclusion

Investigating Hsp-specific T cell responses *in vivo* and *in vitro* is of great interest to examine the effect of Hsp administration on T cell activation and differentiation. Although there are Hsp-specific cell lines that have been generated after long *in vitro* culture (10, 20), as well as Hsp-specific T cell hybridomas (9), these cells lack the properties of primary T cells. A huge disadvantage of these cells is that they lack the ability to differentiate from naive cell to effector or Treg and can therefore only be used for qualitative analysis of Hsp recognition. Several studies have shown that the immunomodulatory effect of Hsp administration (being immunization, intranasal administration, or oral administration) is due to the activation of Hsp-specific Tregs (7, 21–23). However, little is still known about the function of these cells. For instance, it is difficult to study Tregs *in vitro*, since these cells require more than peptide stimulation alone (e.g., growth factors like IL-2 and/or TGF-β) for their expansion and differentiation (24, 25), in comparison to immortalized T cell lines. Eventually, one would like to study primary antigen-specific T cells; however, these are only a minor population within the total population of T cells. Therefore, a TCR transgenic mouse is a valuable tool to obtain larger quantities of antigen-specific T cells. As a result, we set out to generate a TCR transgenic mouse with Hsp70 peptide-specific T cells to establish a tool to study the activation, differentiation, and suppressive capacity of Hsp-specific T cells.

In this study, we have successfully cloned an mB29b-TCR hybridoma with specificity for the Hsp70 peptide mB29b. Due to sequence homologies of Hsp70 family members, this hybridoma can recognize self-Hsp peptides mB29a and mB29b, and the mycobacterial Hsp70 peptide B29. Recognition was MHC class II dependent, and all APCs tested were capable of activating the mB29b-TCR hybridoma (Figures 1 and 2). Next, the TCRα and TCRβ chain were isolated and cloned into TCR expression vectors, which were electroporated into TCR^−^ T cells, which showed peptide-specific activation upon electroporation of the construct (Figure 3). Although the hybridomas did show a response to mB29a (Figures 1 and 2), the TCR^−^ cells in which the expression vectors from hybridoma LHEP4 were transfected showed little response to this peptide as measured by IL-2 production. This can be explained by the fact that hybridomas produce much larger amounts of IL-2 in response to antigen-specific stimulation compared to the TCR^−^ cells. In addition, the mB29a peptide was found to be a less strong agonist than the founding peptide mB29b when used to stimulate splenocytes from the mB29b TCR Tg mouse, most likely due to the two amino acids difference (Table 1).

After linearization of the mB29b-TCR plasmids, the TCRα and TCRβ chain were injected pronuclear into mouse oocytes. Two founders were born that carried both vectors. PBLs from one of the mB29b-TCR transgenic founders showed mB29b-specific activation, as well as cross-recognition to the Hsp70 peptide B29 (Figure 4). This founder (founder 2) was mated with Balb/c mice and offspring was screened for bearing the mB29b-TCR. Splenocytes were stimulated with Hsp peptides and showed similar peptide recognition as the founder did. FACS analysis revealed that the distribution of CD4^+^ and CD8^+^ T cells in thymus and spleen of the mB29b-TCR transgenic were slightly different to that of littermates (Figure 5). Differences in CD4^+^ and CD8^+^ T cell distribution in TCR transgenic mice compared to non-transgenic littermates is due to the transgenic background in which formation of T cells is altered. This is considered normal for a TCR transgenic mouse (26, 27), since these mice will have selective development of αβTCR T cells in the thymus. The distribution of TCR transgenic cells could be seen in histology of the thymus (Figure 6). Although, in general, the T cell distribution in the tissue sections was comparable between the mice, a decreased CD4^+^CD25^+^FoxP3^+^ population was observed in naive mB29b TCR transgenic mice by flow cytometry, comparable to several other TCR transgenic mice (28, 29). Furthermore, the amount of proliferated cells was altered in lymphoid tissues from the mB29b-TCR transgenic mouse compared to littermates. The mB29b-TCR Tg mice showed a decrease in KI-67^+^ cells, which might be due to the fact that mB29b is a self-peptide which causes self-regulation. The data do indicate that tissue morphology is normal in the mB29b-TCR transgenic mouse. Furthermore, no spontaneous autoimmune disease was observed in these young TCR transgenic mice.

With this new TCR transgenic mouse, we are now able to study the properties of naive T cells differentiating and proliferating into effector and Tregs. Furthermore, we now hope to elucidate the mechanism of Hsp70-mediated T cell regulation without prior Hsp70 immunization and its associated non-specific immune activation.

## Author Contributions

MJ and MH designed and performed experiments, analyzed data, wrote the paper, and approved the submitted version. PK designed and performed experiments, analyzed data, and approved the submitted version; AH performed experiments, analyzed data, and approved the submitted version; RZ designed experiments, analyzed data, commented on the manuscript at all stages, and approved the submitted version; WE commented on the manuscript at all stages and approved the submitted version; FB designed experiments, commented on the manuscript at all stages, and approved the submitted version.

## Conflict of Interest Statement

The authors declare that the research was conducted in the absence of any commercial or financial relationships that could be construed as a potential conflict of interest.
